# Normal sonographic anatomy of the abdomen of coatis (*Nasua nasua* Linnaeus 1766)

**DOI:** 10.1186/1746-6148-9-124

**Published:** 2013-06-23

**Authors:** Rejane G Ribeiro, Ana Paula A Costa, Nathália Bragato, Angela M Fonseca, Juan CM Duque, Tales D Prado, Andrea CR Silva, Naida C Borges

**Affiliations:** 1 Pos graduation degree in Animal Science, School of Veterinary Medicine, Federal University of Goiás, Goiânia, Brazil; 2 Medical resident in Veterinary Imaging Diagnostic, School of Veterinary Medicine and Animal Science, Federal University of Goiás, Goiânia, Brazil; 3 Medical resident in Veterinary Anesthesiology, School of Veterinary Medicine and Animal Science, Federal University of Goiás, Goiânia, Brazil; 4 Department of Veterinary Medicine, Anesthesiology Service, School of Veterinary Medicine and Animal Science, Federal University of Goiás, Goiânia, Brazil; 5 Department of Veterinary Medicine, Imaging Diagnostic Service, School of Veterinary Medicine and Animal Science, Federal University of Goiás, Goiânia, Brazil

## Abstract

**Background:**

The use of ultrasound in veterinary medicine is widespread as a diagnostic supplement in the clinical routine of small animals, but there are few reports in wild animals. The objective of this study was to describe the anatomy, topography and abdominal sonographic features of coatis.

**Results:**

The urinary bladder wall measured 0.11 ± 0.03 cm. The symmetrical kidneys were in the left and right cranial quadrant of the abdomen and the cortical, medullary and renal pelvis regions were recognized and in all sections. The medullary rim sign was visualized in the left kidney of two coatis. The liver had homogeneous texture and was in the cranial abdomen under the rib cage. The gallbladder, rounded and filled with anechoic content was visualized in all coatis, to the right of the midline. The spleen was identified in the left cranial abdomen following the greater curvature of the stomach. The parenchyma was homogeneous and hyperechogenic compared to the liver and kidney cortex. The stomach was in the cranial abdomen, limited cranially by the liver and caudo-laterally by the spleen. The left adrenal glands of five coatis were seen in the cranial pole of the left kidney showing hypoechogenic parenchyma without distinction of cortex and medulla. The pancreas was visualized in only two coatis. The left ovary (0.92 cm x 0.56 cm) was visualized on a single coati in the caudal pole of the kidney. The uterus, right adrenal, right ovary and intestines were not visualized.

**Conclusions:**

Ultrasound examination of the abdomen of coatis may be accomplished by following the recommendations for dogs and cats. It is possible to evaluate the anatomical and topographical relationships of the abdominal organs together with the knowledge of the peculiarities of parenchymal echogenicity and echotexture of the viscera.

## Background

The coati (*Nasua nasua*) is a member of the Procynidae family of the Carnivorous order [1]. With the exception of Chile [2], this is a specimen exclusive of South America.

It is an animal that easily adapts and socializes with humans; therefore, most studies related to this species refer to the reproductive area, aiming to control population in reserves and zoos, or with their ecological role of seed dispersal [3]. As these animals can also be reservoirs for pathogens of diseases such as leishmaniasis, rabies and distemper, there is the concern of transmission of some of these diseases to human populations or domestic animals [4].

Thus, there is a need for detailed information on aspects related to diseases affecting the coatis and for veterinarians to deepen their knowledge of available diagnostic imaging aids. There is no doubt that the science-oriented studies of anatomy and physiology are the basis for the interpretation of imaging studies, as well as for the success of clinical and surgical procedures [5].

Ultrasound is a widespread diagnostic imaging modality in veterinary medicine that provides real time information about the architecture and ultrasonographic characteristics of the organs, assisting in the identification of the physiological conditions and abnormalities in various organs and diseases. Furthermore, ultrasound is portable, does not emit radiation and usually does not require general anesthesia, except in wildlife animals. It can be used in clinics, laboratories and field, but its use is still limited in wild animals, due to the limited knowledge of the topography and ultrasound anatomy of their organs [6].

According Peixoto et al. [6] and Nyland et al. [7] it is essential that the sonographer has an extensive knowledge of anatomy, physiology and pathophysiology of the studied species, because knowledge of the topography and ultrasound anatomy of organs to be examined are prerequisites for an ultrasound, given that the accurate interpretation depends directly on the differentiation between normal and abnormal structures.

The aim of this study was to identify a technique for abdominal ultrasonographic examination of the coatis and to describe the normal ultrasonographic anatomy of its abdominal organs.

## Results

### Urinary Bladder

**Figure 1:**
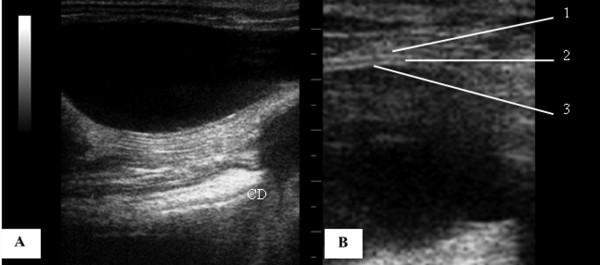
**Transverse sonogram of the urinary bladder of an adult male Coati. A**) Urinary bladder filled with anechoic urine content in usual topography, ventral to the descending colon (CD). **B**) Magnified image for detail of the urinary bladder wall, 1 – serosal layer (hyperechoic), 2 - muscular layer (hypoechoic), 3 - submucosal layer (hyperechoic).

The urinary bladder (UB), filled with urine was identified as an oval and anechoic structure, at the caudal abdomen, ventral to the descending colon (Figure 1A). At the urinary bladder wall two thin hyperechoic lines separated by a hypoechoic line were seen, corresponding a hyperechoic serosa with a perivascular fat interface, hypoechoic muscularis and a hyperechoic line of submucosa paralleling with mucosal interface (Figure 1B). The wall thickness was 0.11 ± 0.03 cm (0.11 cm minimum and maximum 0.17 cm).

### Uterus and ovaries

**Figure 2:**
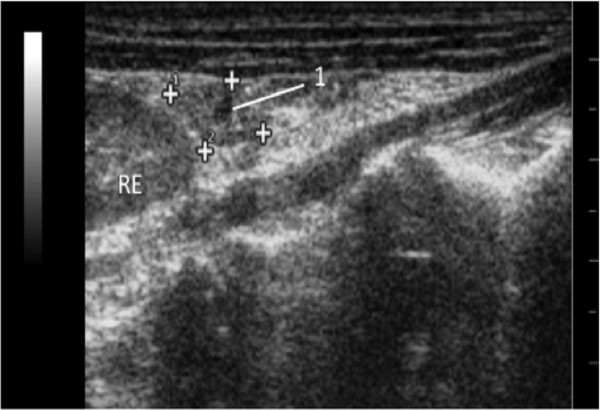
**Transverse sonogram of the left ovary (between cursors) of an adult Coati.** An central circular anechoic area (1) indicates a cyst. RE, left kidney.

The body and uterine horns were not visualized. Only the left ovary, in one coati, was visualized in the caudal pole of the left kidney, as a hypoechoic structure, measuring 0.92 cm width and 0.56 cm in length. The parenchyma of the ovary revealed an anachoic circular area, indicative of ovarian cyst or follicle (Figure 2).

### Kidneys

**Figure 3:**
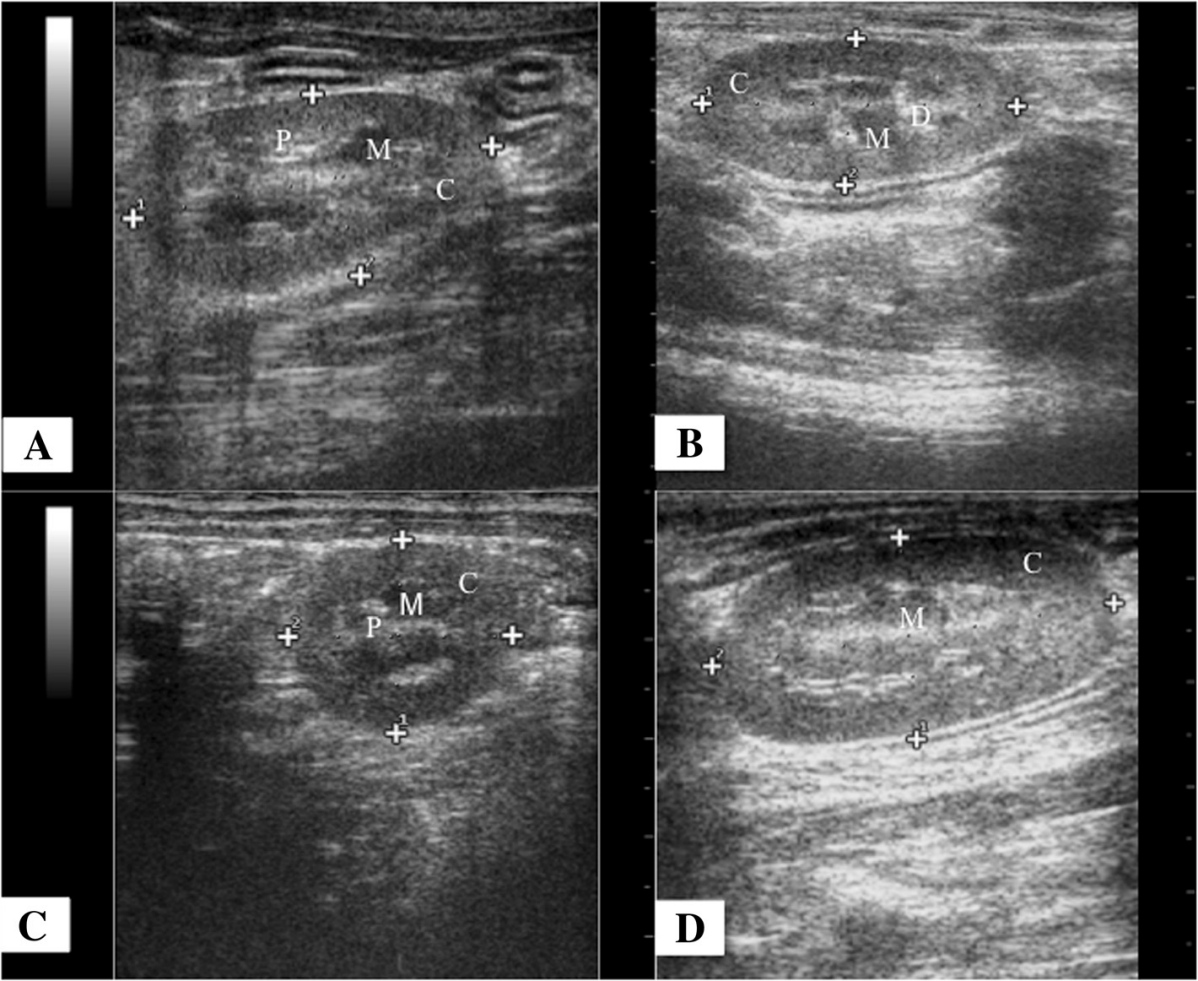
**Sonogram of the kidneys of an adult Coati.** Kidney in sagittal (**A**), dorsal (**B** and **D**) and transverse (**C**) planes. Observe the indication of the cortical (C), medullary (M), renal pelvis (P) and diverticulum (D).

The kidneys were evaluated in the right and left cranial quadrant of the abdomen, just below the second last rib, with the right kidney being in a position slightly more cranial than the left. The sagittal (Figure 3A) and dorsal (Figure 3B) planes showed that both kidneys had an oval format, regular contours and slightly hyperechoic renal capsule, in cross section the kidneys appear with a rounded shape (Figure 3C).

Three distinct regions were visualized in all planes, an external one with a homogeneous and hyperechoic appearance indicating the cortex, the intermediate hypoechoic to anechoic area indicating the renal medulla and a central hyperechoic region indicating the area of the renal pelvis (Figure 3A, B, C, D). On the dorsal plane the diverticula were also identified (Figure 3B).

The values obtained in the measurement of length, height, width and thickness of the coati’s kidney cortical of are shown in Table 1.

**Table 1:** Means, standard deviation, minimum and maximum values of right and left kidney sonographic measurements in seven adults Coatis (Nasua nasua) raised in semi-captivity

**Planes**	**Dimensions**	**Kidney**	**Mean**	**Standard**	**Minimum**	**Maximum**
	**(cm)**		**Values**	**Deviations**	**Values**	**Values**
Transverse	Width	Right	1.82	0.23	1.51	2.20
		Left	1.81	0.26	1.30	2.14
	Cortical thickness	Right	0.39	0.06	0.33	0.47
		Left	0.40	0.02	0.37	0.45
Dorsal	Length	Right	3.06	0.19	2.81	3.39
		Left	3.03	0.26	2.83	3.47
	Height	Right	1.62	0.15	1.36	1.85
		Left	1.52	0.16	1.35	1.76

### Liver and gallbladder

**Figure 4:**
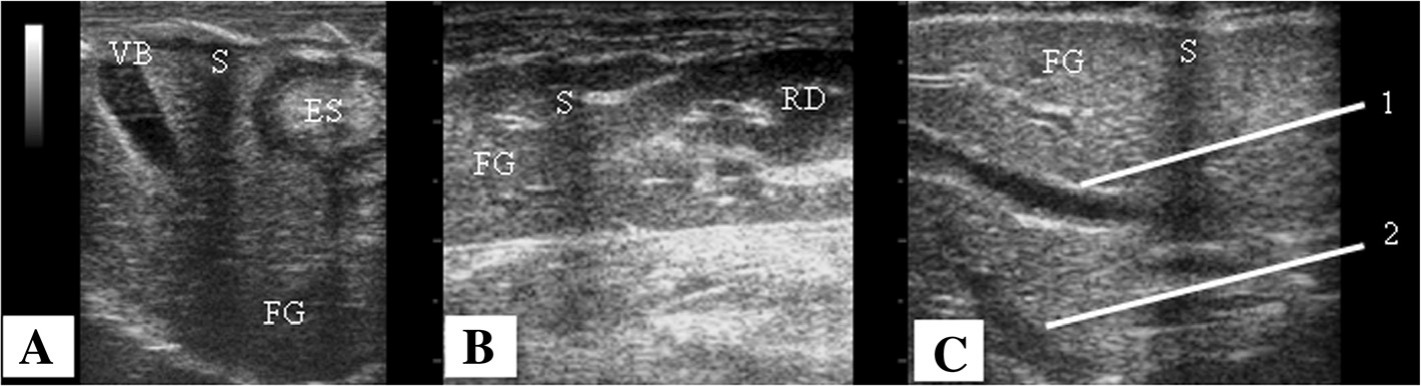
**Sonogram of the liver in longitudinal section an adult Coati. A**) Anatomic relationship of hepatic sublobe and stomach. Gallbladder is visible having a teardrop shape, filled with anechoic homogeneous content and a thin hyperechoic wall. **B**) Anatomic relationship of the caudate sublobe with the right kidney. **C**) Identification of the portal vein with hyperechoic walls (1) and hepatic veins with isoechoic walls (2). **A**) Acoustic shadowing due to the presence of a rib can be seen in all images (S).

The liver was seen occupying the entire length of the cranial abdomen inside the rib cage and in close contact with the diaphragm. The topographical relationships of the liver were the caudate lobe in contact with the right kidney, stomach centrally (Figure 4A, B), and to its left, the spleen. The liver parenchyma had moderate echogenicity and mildly heterogenous echo texture.

The branches of the portal veins were visualized with hyperechoic walls and hepatic veins, characterized by numerous anechoic tubular structures (Figure 4B). The gall bladder was observed in all coatis to the right of the midline, as a rounded structure filled with anechoic homogeneous contents with thin hyperechogenic wall (Figure 4A).

### Spleen

**Figure 5:**
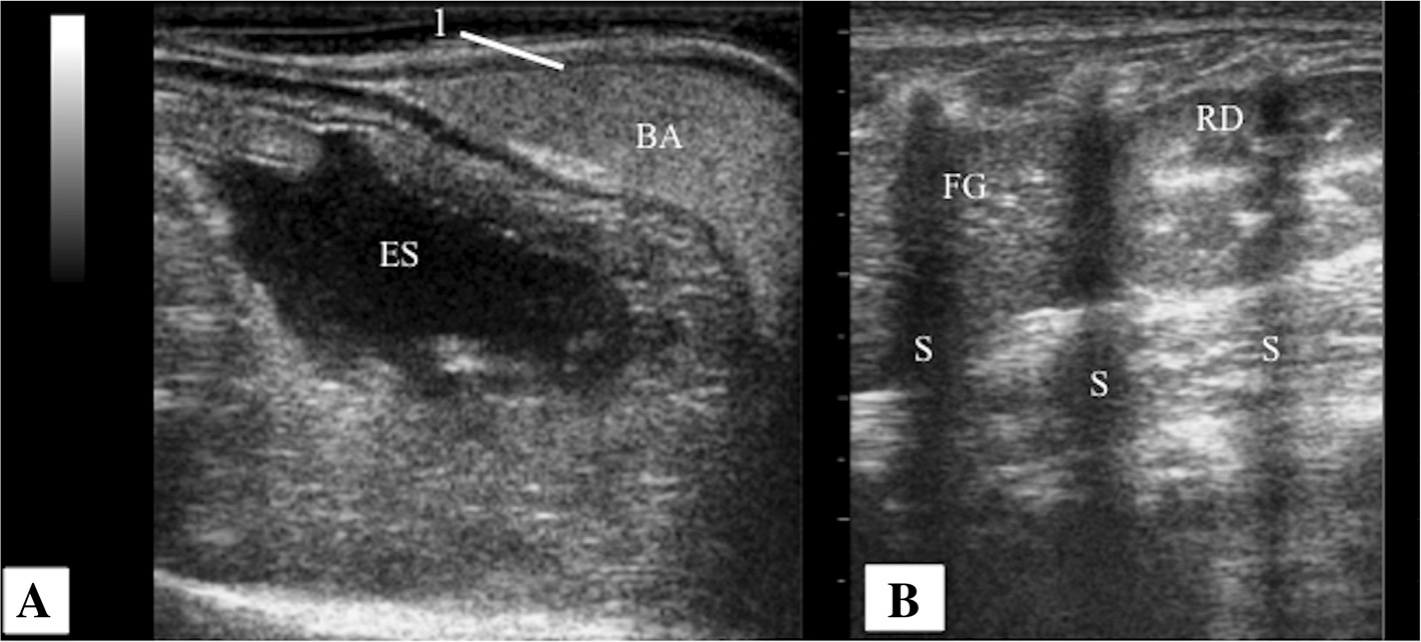
**Sonogram of the spleen in longitudinal section an adult Coati.** The hyperechoic capsule (1) and the cranial end to the greater stomach curvature were observed and the echogenicity relationship between the hyperechoic spleen (**A**) with the less echogenic renal cortex, and the liver which is isoechoic in relation to the renal cortex (**B**). BA, spleen, ES, stomach, FG, liver, RD, right kidney. Acoustic shadowing due to the presence of ribs (S).

The spleen, falciform or triangular in shape, was located in the left cranial abdomen under the rib cage, following the great stomach curvature and ventral to the left renal capsule surrounded by a thin hyperechoic capsule (Figure 5A). The splenic parenchyma observed as being homogeneous and hyperechoic when compared to the liver and the left renal cortex (Figure 5A, B). The splenic vein was not visualized.

### Adrenal gland

**Figure 6:**
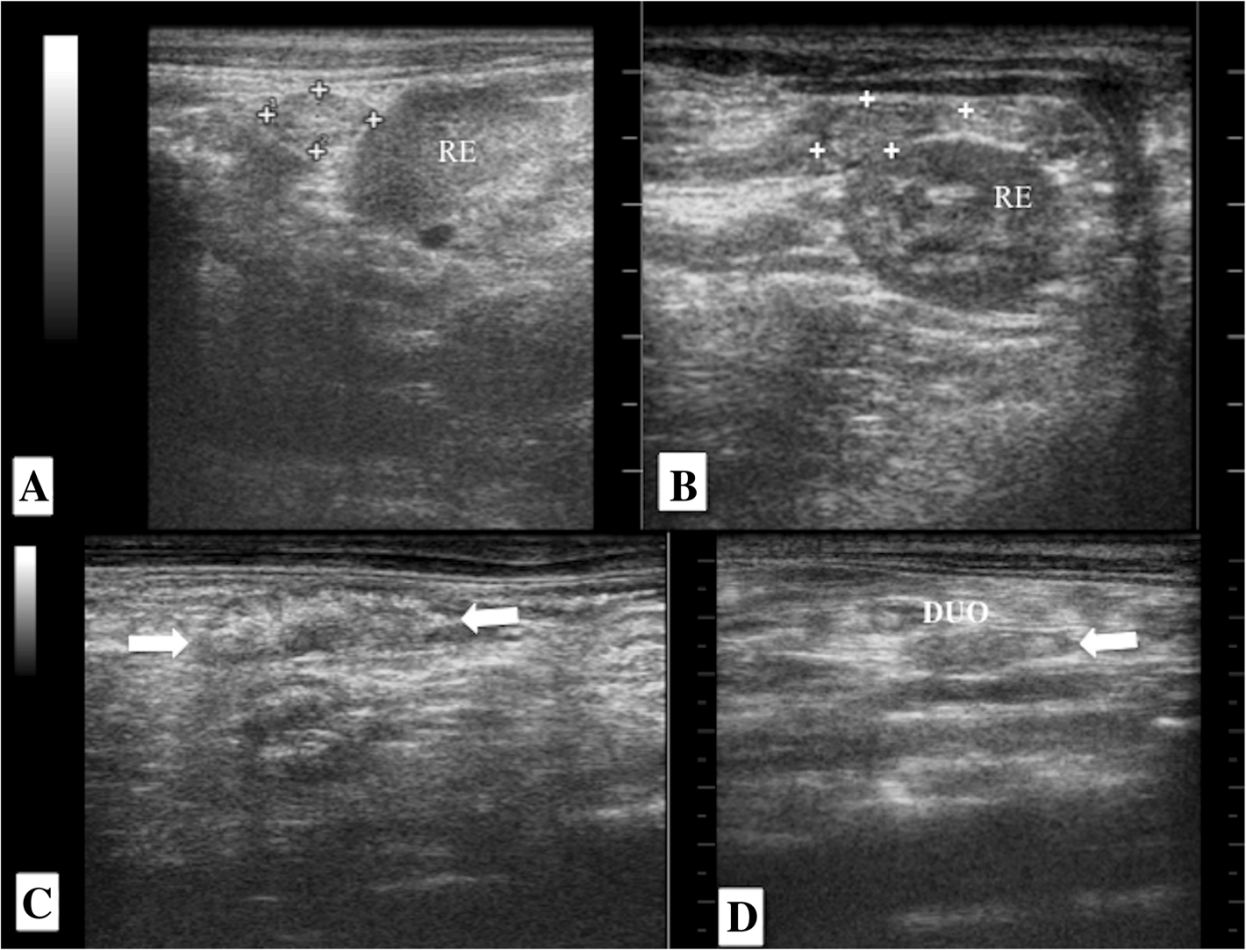
**Sonogram of the adrenal and pancreas in an adult Coati. A**) Left adrenal left in longitudinal section (between cursors), note the relationship to the cranial pole of the left kidney, **B**) Left adrenal in transverse section (between cursors), keeping the anatomical relationship with the left kidney in transverse section. RE, left kidney. Pancreas, hypoechoic in relation to adjacent tissues. Between arrows in **C**) and adjacent to duodenum (DUO) in **D**).

Only the left adrenal was seen in five of seven coatis evaluated. They were located in the cranial pole of the left kidney, oval shaped, having a hypoechoic parenchyma in relation to the surrounding tissues, without distinction of cortex and medulla (Figures 6A and 6B). The measurement of the gland was found to be 0.92 ± 0.52 cm long (range 0.50-1.68 cm) and 0.52 ± 0.24 cm width (range 0.38-0.8 cm).

### Pancreas

In two coatis the right lobe of the pancreas was visualized housed in the right cranial abdomen, dorso-medial to the descending duodenum. In one animal the pancreas was isoechoic to the adjacent tissues and in the other coati the pancreas body was hypoechogenicity in relation to adjacent tissues (Figures 6C and 6D).

### Stomach and intestine

**Figure 7:**
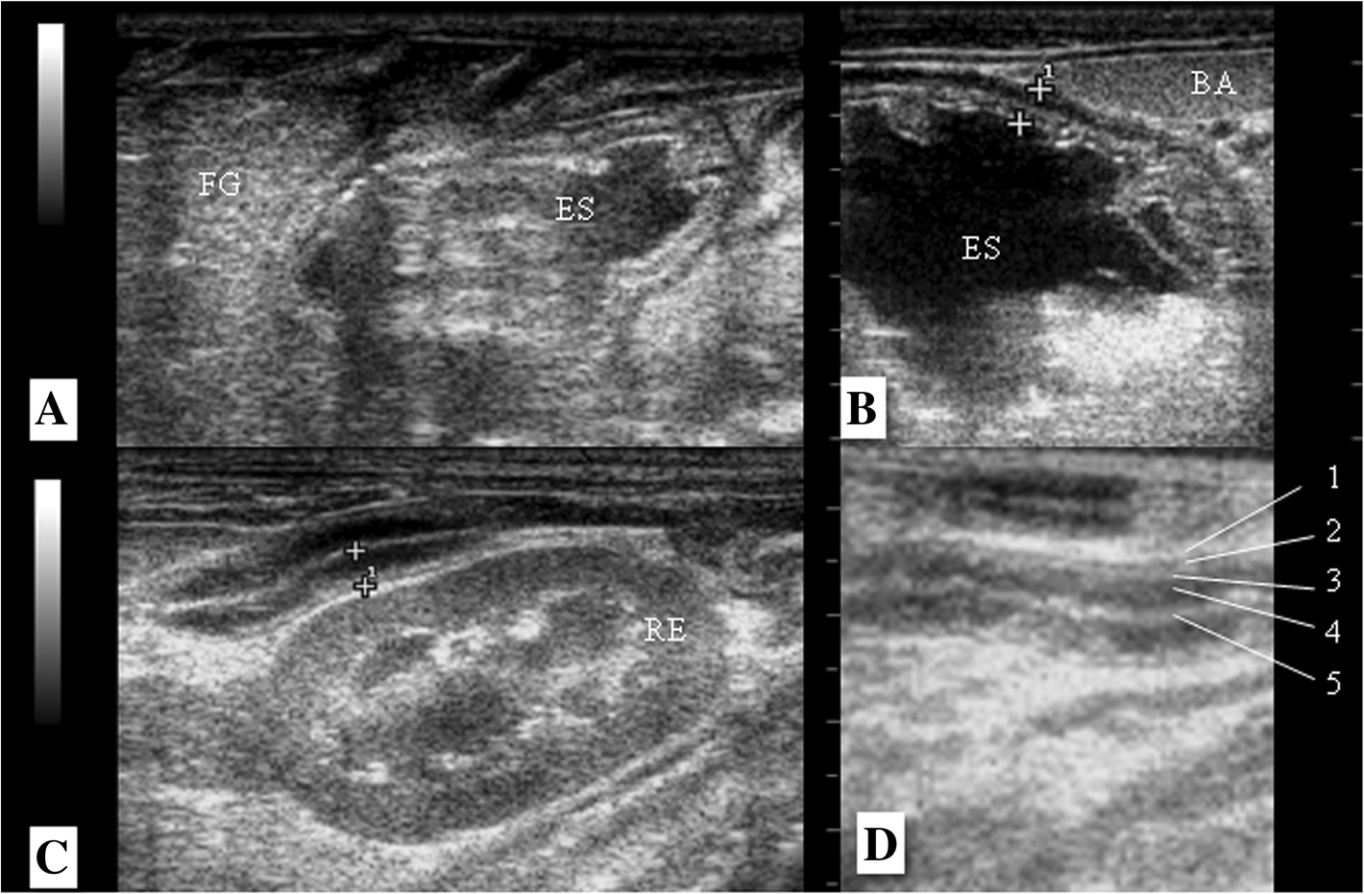
**Sonogram of the gastrointestinal tract in an adult Coati.** Anatomical relationship of the stomach with the liver (**A**) and spleen (**B**). Measurement of the intestinal wall in the longitudinal section shown between cursors (**C**). Measurement of the gastric wall in longitudinal section indicated between cursors (**D**). In detail, the layers of the intestinal wall, 1- serosal and subserosal layer (hyperechogenic), 2 – muscularis (hypoechogenic), 3 - submucosa (hyperechogenic), 4 - mucosa (hypoechogenic), 5 - lumen (hyperechogenic). FG, liver, ES, stomach, BA, spleen, RE, left kidney.

The stomach was located in the left cranial abdomen, limited cranially to the liver (Figure 7A) and caudolaterally to the spleen (Figure 7B). The wall of the stomach and intestines had five layers, the serosa and subserosal (hyperechoic), the muscularis (hypoechoic), the submucosa (hyperechoic), the mucosa (hypoechoic) and the lumen (hyperechoic) (Figure 7D). The gastric wall and the intestinal wall thicknesses are listed on Table 2.

**Table 2:** Mean, median, maximum and minimum values, standard deviations and coefficient of variation (CV) of gastric and intestinal wall sonographic measurements in seven adults Coatis (Nasua nasua) raised in semi-captivity

**Wall thickness**	**Mean**	**Median**	**Maximum**	**Minimum**	**Standard**	**CV**	**P**
	**(cm)**	**(cm)**	**(cm)**	**(cm)**	**Deviations**	**(%)**	Value*
Gastric	0.31	0.30	0.64	0.13	0.11	50.76	0.71
Intestinal	0.21	0.20	0.34	0.15	0.06	30.58	0.15

*Shapiro-Wilk test (p > 0,05).

## Discussion

Chemical restraint is essential with sedation or even general anesthesia to perform the ultrasound examination in wild animals [8]. For dogs and cats, chemical restraint is only used in interventional ultrasound procedures such as aspiration biopsy, drainage of cysts and paracentesis [9].

The coatis were sedated with an association of pre-anesthetic ketamine, midazolam and meperidine. The association of ketamine with midazolam was consistent with the description of anesthetic protocols for coatis reported by Gregores [10], Kollias et al. [11] and Tan et al. [3]. However, the opioid meperidine was also used to promote balanced anesthesia, producing a reduction in the dose of each drug used and in the occurrence of adverse effects [12].

Anesthetic induction was accomplished by using a face mask with isoflurane as suggested by Catwell [13], and the anesthetic was administered through a gas circuit without rebreathing. As the induction the maintenance of the anesthesia was performed with isoflurane, wich, according to Denver [14], is the agent of choice for racoons. The dose of each drug were based on reports in the literature of both wild animals [15], dogs and cats [16], agreeing with the statements of Schoemaker et al. [17], wich is, that the chemical restraint, the techniques and anesthetics protocols applied to mammals of the Procynidae family in captivity are similar to those used for domestic dogs and cats.

Ultrasound examination was carried out with the coatis placed in the supine position after an extensive shaving of the ventral abdomen, as recommended for dogs and cats by Carvalho [18] and Mattoon et al. [19], for performing sonographic examination. With the transducer selected at a 7.5 MHz frequence, depth of seven inches and 79% gain was possible to obtain an adequate image resolution for the coatis abdominal study. These choices were according to Mattoon et al. [19] that recommended a 7.5 MHz frequency for ultrasound examinations in small dogs and cats, and with Cheida et al. [20] that classified the coatis as small to medium sized animals, weighing between 2.7 to 10 kg.

The abdomen of the coatis was assessed methodically starting with the bladder, and surrounding areas, following to left kidney, spleen, left adrenal, left ovary, stomach, liver, gallbladder, right kidney, right adrenal, right ovary, pancreas and intestines. The sequence was employed for the abdominal ultrasound contrary instructions of Cortassi [19] and Penninck et al. [21], which proceed abdominal scans in the order of liver, spleen, stomach, duodenum, pancreas, kidney, adrenal, bladder, prostate and lymph nodes sublombares, followed by scanning in the intestinal tract and the remaining additional abdominal lymph nodes.

Only four coatis showed the urinary bladder (BU) with adequate filling for evaluation. The wall of the urinary bladder has been identified as two hyperechogenic lines separated by hypoechogenic a line (Figure 1B) as described for dogs and cats [22]. The average wall thickness of the urinary bladder of coatis varied from 0.11 cm to 0.17 cm, similar to the measures described for dogs (0.1 cm to 0.2 cm) and cats (0.13 cm to 0.17 cm) [7]. When filled with urine, the urinary bladder was identified as an anechoic structure, oval shaped in the caudal abdomen, ventral to the descending colon (Figure 1A).

The uterus was not visualized in any coati. According to Jarreta [23] and Mattoon [24] in domestic species such as dogs and cats, the uterine assessment is facilitated in pregnant and in estrus animals or those with a uterus disease. Corroborating this assertion, Chittick et al. [25] diagnosed, through ultrasound, a captive coati *(Nasua nasua*), who used contraceptive implant, with pyometra. Thus, we can say that in the present study, no females had abnormal uterus perceptible to the sonographic examination.

According Jarreta [23], the ovaries of domestic carnivores are small and difficult to visualize by having similar echogenicity to the adjacent tissues. Furthermore, according Jarreta [23] and Mattoon et al. [24] the location of the right ovary, between the duodenum and the lateral wall of the abdomen, makes it difficult to identify, because the presence of air causes reverberation artifect that prevents the passage of sound and image formation of the ovary.

In this study only the left ovary was visualized, possibly due to its location between the duodenum and the lateral wall of the abdomen like described Jarreta [23] and Mattoon et al. [24]. The left ovary contained a small anechoic area, suggesting the presence of cyst or follicle (Figure 2), which probably contributed to their identification. Beregi et al. [26] reported that ultrasound is effective in the evaluation of ovarian diseases in guinea pig (*Cavia porcellus*), in which they diagnosed cysts larger than 1.5 cm in diameter.

The kidneys of coatis were oval in shape, had regular contours and hyperechoic capsule (Figure 3B). Furthermore, it was observed a hypoechogenic cortical, an anechoic medullary, and a hyperechogenic pelvis with well defined corticomedullary relationship (Figure 3). These findings resemble those observed in dogs and cats [27-29], in cheetahs (*Cinonyx jubatus*) [30] and in capuchin monkeys (*Cebus apella*) [31].

In contrast, Wagner et al. [32] evaluating the kidneys of marmoset, found that in this species there is a poor corticomedullary distinction and the cortex is hyperechogenic. According to Vac [22] these descriptions observed in marmosets indicate glomerular or interstitial nephritis in dogs and cats. Extrapolating this opposition results to those obtained with the coatis, it can be seen that the interpretation of the findings in a sonographic examination is entirely related to the knowledge of ultrasonographic anatomy and that the unawareness can lead to misdiagnosis.

As there is little variation in weight between coatis, as observed in cats, the linear measurements are more useful than in dogs in which the measurement is made subjectively because of the variety of size and body volume in animal of equal weight [27]. According to Vac [22] measures are more appropriate to compare the kidneys of animals that must be symmetrical, as seen in coatis (Table 1).

The medullary rim sign was identified in two left kidneys of coatis (Figure 3d). According to Vac [22] is not a pathognomonic ultrasound finding of renal disease. However, Mantins and Lamb [33] and Nyland et al. [27] reported that this finding was observed in dogs and cats with kidney disease and in clinically healthy animals, being investigations necessary to determine the causes and significance of this sign. Carsten et al. [30] observed medullary rim sign in 21 of 26 kidneys of healthy cheetahs. These results lead to the fact that is necessary to know the specific ultrasound appearance of the studied system and animal species.

Cranio-medial to left kidney and cranial to the left renal artery we encountered the left adrenal of five coatis (Figure 6). Similarly, Chapman et al. [34] and Nyland et al. [35] emphasized the ease of identifying the left adrenal in dogs and cats compared to the right due to the anatomic location, described for the study of coatis. Moreover, Wagner et al. [32] observed the adrenals of marmosets in an anatomical position similar to that of carnivores, however both adrenals were easily found when employed silicone cushion for the sonographic evaluation.

The adrenal glands of coatis had oval contour (Figure 6) as observed in cats, different from dogs that usually have a resembling peanut-shape the adrenal gland [34]. As for the echogenicity the adrenal glands were found to be hypoechogenic relative to surrounding tissue and it was not possible to distinguish between cortical and medullary regions (Figure 6). Santos et al. [29] reported this feature in the parenchyma of the adrenal glands of puppies and kittens, unlike adult dogs and cats which is commonly visualized corticomedullary distinction according to Carvalho et al. [34] and Nyland et al. [35]. Despite the find of this study, to support this definition, we suggest that a greater number of coatis must be evaluated, considering that sample of adult animals used in this study was small.

The liver of coatis is located entirely below the rib cage, being the left lobe to the left of the midline, the quadrate in the middle plane, the right lobe to the right of the midline and the caudate process of caudate lobe in contact with the cranial pole the right kidney (Figure 4B). The cranial edge of the liver is bounded by the diaphragm, a hyperechoic curvilinear structure, and the caudal margin by the stomach, similar as seen in dogs and cats (Figure 4A).

The portal veins have hyperechoic walls unlike the hepatic veins, which were isoechoic to the surrounding tissue (Figure 4C), features also observed in domestic animals by Mamprim [36] and Nyland et al. [37]. The gallbladder, round or teardrop shaped and with an anechoic content (Figure 4A), preserved the characteristics described in quotations from studies in dogs and cats [37]. Interestingly, Wagner et al. [32] found that in marmosets the gallbladder has a bilobed silhouette, which is considered naturally found in domestic cats as mentioned Mamprim [36] and Nyland et al. [37]. Again, the anatomical knowledge proved to be crucial for the interpretation of sonographic findings, and should be followed in this coatis study.

In this study, the spleen was easily visualized due to its size and superficial location, without interference of gas from the intestinal contents. Our findings differ from Alves et al. [31] who found that the spleen was the most difficult organ more to be evaluated in capuchin monkey due to its small size in this species, allowing the evaluation of the spleen only in one animal among the ten studied. In coatis, the spleen was falciform or triangular shaped, with thin echogenic capsule (Figure 5A), and of a homogeneous echo texture similar to the spleen of the dog and cat, according to the descriptions of Santos [29] and Tannouz et al. [38].

Analysing the relationship of echogenicity of the triad, spleen, liver and kidney, it was found that in all coatis presented the spleen was more echogenic than the liver, and the liver was isoechoic to the renal cortex (Figure 5B). These characteristics are also reported in domestic carnivores by Tannouz et al. [38] and Nyland et al. [39] and in cheetahs by Carsten et al. [30]. Contrary to this description, all healthy marmosets evaluated by Wagner et al. [32] presented the spleen more hypoechoic than the liver and renal cortex.

When the transducer was craniomedial directed to the spleen we identified the stomach, caudal located to the hepatic parenchyma (Figure 7a) and cranial to the left kidney, similar to what is described for dogs and cats by Froes [40]. The gastric and intestinal wall was easily evaluated and presented five layers with different echogenicity (Figure 7C, D) as also verified by Mattoon et al. [19] in domestic carnivores and Wagner et al. [32] in marmosets. The thickness of the stomach (Figure 7D) and intestine (Figure 7C) was also similar to that described for dogs and cats by Mattoon et al. [19] and Froes [40].

The pancreas was observed in only two coatis. The parenchyma presented isoechoic to the surrounding tissue (Figure 6C) and slightly hypoechoic (Figure 6D). These differences of echogenicity also were observed in healthy dogs and cats [19]. This limitation for identification can be justified if we consider the remarks of Zardo et al. [41] about the fact that even though the method of choice for assessing the pancreatic body in veterinary medicine is the sonographic examination, it has limitations regarding the quality of the ultrasound equipment, experience of the sonographer, similarity with echogenicity of the surrounding tissues, interfering of gases and food contents of the gastrointestinal tract. Wagner et al. [32] justify that they could not visualized of the pancreas in a study with marmosets because the limited time of the examination (30–40 minutes).

Thus, it is believed that the interpretation of ultrasound examinations is directly related to the knowledge of topographic anatomy, as well as the sonographic distinction of the various abdominal organs, of the various animals’ species. Considering all these aspects, the experienced sonographer may assist clinicians and surgeons in diagnostic and prognostic interpretations.

## Conclusions

Ultrasound examination of the abdomen of coatis may be accomplished by following what is recommended for dogs and cats. It is effective to evaluate the anatomical and topographical relationships of the abdominal organs together with the knowledge of the peculiarities of parenchymal echogenicity and echo texture of the viscera.

## Methods

This study was conducted under the authorization of the Brazilian Institute of Environment and Natural Renewable Resources (Instituto Brasileiro do Meio Ambiente e dos Recursos Naturais Renováveis - IBAMA), under license number 014/2011 and was approved by the Ethics Committee on Animal Experimentation of Federal University of Goiás registration number 118/11.

The study was conducted in seven adults coatis considered clinically healthy, four females and three males, with average weight of 2.67 ± 0.54 kg. The animals were from the Center for Screening of Wild Animals (Centro de Triagem de Animais Silvestres - CETAS), an IBAMA agency, located in Goiânia, Goiás, Brazil. The exams were realized in the Veterinary Hospital of the Veterinary and Animal Science School, Federal University of Goiás.

As recommended, the animals underwent a 12 hour fasting. At the Veterinary Hospital, coatis were sedated with 15 mg/kg of ketamine hydrochloride (Ketamine Agener 10% União Química Farmacêutica Nacional S/A, Embu-Guaçu- SP, Brazil) associated with 0.2 mg/kg of midazolam (Dormine 0.5% Cristália Produtos Químicos Farmacêuticos Ltda, Itapira- Lindóia- SP, Brazil) and 5 mg/kg meperidine hydrochloride (5% Dolosal, Cristália Produtos Químicos Farmacêuticos Ltda, Itapira- Lindóia- SP, Brazil), all in the same syringe, intramuscularly.

The intravenous catheter of 22 gauges was placed in the cephalic or femoral vein for drug administration and fluid-Care with sterile Ringer's lactate at a dose of 10 ml/kg/hour (Ringer's Lactate. Equiplex Indústria Farmacêutica Ltda., Aparecida de Goiânia-Go, Brazil). Afterwards, an anesthetic protocol was established, in which the coatis were induced with oxygen (O2) mask at 100% and a four liters per minute (L/min) flow, with the vaporizer calibrated to provide 3.5% isoflurane (Isoflurano, Instituto Biochimico Indústria Farmacêutica Ltda, Itatiaia-RJ, Brazil) in a rebreathing circuit of gases, of the Baraka type. and calibrated vaporizer melhor tirar.

After induction, the animals were intubated with an endotracheal tube, internal diameter of three and a half or four millimeters, and maintained under spontaneous ventilation with a 1.0 inspired oxygen fraction (FiO_2_) and a 300 mL/kg volume of fresh gas.

### Sonographic evaluation

For the sonographic evaluation the hair of the abdominal ventral region, from the costal arch to the inguinal region, of the coatis was clipping. Then a heated acoustic gel was applied to the animals’ skin to prevent hypothermia and to assist in acoustic contact between the transducer and the patient. The animals were positioned a supine position to perform the sonographic evaluation with the ultrasound equipment My Lab™ 30 Vet (The Esaote Group, Genova, Italy) coupled to the multifrequency linear transducer (7.5 MHz to 12.0 MHz), selected for 7.5 MHz frequency and a 79% gain.

The positioning of the transducer to the achievement of the sagittal and transverse of the urinary bladder, spleen, liver, gall bladder, adrenal, ovary, pancreas and gastrointestinal tract, as well as the evaluation of contour, margin, size, texture and echogenicity of these organs followed the instructions Carvalho [18].

The kidneys were evaluated in the longitudinal, transverse and dorsal planes to determine the contour, margin, echo texture and echogenicity following the recommendations of Vac [22]. The measurements were performed on images obtained with the dorsal and transverse planes. According Nyland et al. [27], from the dorsal plane the measures of length (distance between the cranial and caudal poles) and height (distance between the ventral and dorsal surfaces) was determined. From the transversal plane the kidney width (distance between the dorsal and ventral surfaces and cortical thickness (distance between the outer edge of the kidney and renal capsule) were measured.

### Statistical analysis

Descriptive statistical analysis was performed for presentations of the measurements of abdominal organs evaluated. The Shapiro-Wilk test was used to determine the normal range of wall thickness of stomach and intestine. The same test was not applied in other organs studied, since these were not visualized at all coatis (n < 5).

## Competing interest

The authors declare that they have no competing interest.

## Author's contributions

JCDM and AMF anesthetized the animals. RGR, NCB, APC and NB did the sonographic examinations. RGR and NCB have written the manuscript. RGR, APC, NCB, TDP, ACRS helped in the review. All authors read and approved the final manuscript.
